# Disentangling the acute subjective effects of classic psychedelics from their enduring therapeutic properties

**DOI:** 10.1007/s00213-024-06599-5

**Published:** 2024-05-14

**Authors:** Mazen A. Atiq, Matthew R. Baker, Jennifer L. Vande Voort, Maxemiliano V. Vargas, Doo-Sup Choi

**Affiliations:** 1 Department of Laboratory Medicine and Pathology, Mayo Clinic, Rochester, MN USA; 2 Department of Molecular Pharmacology and Experimental Therapeutics, Mayo Clinic, 200 First Street, SW, Rochester, MN 55905 USA; 3 Department of Psychiatry and Psychology, Mayo Clinic, 200 First Street, SW, Rochester, MN 55905 USA; 4 Institute for Psychedelics and Neurotherapeutics, University of California, Davis, Davis, CA USA

**Keywords:** Psychedelics, Acute subjective effects, Psilocybin, Classic psychedelics, 5-HT2A, Major depressive disorder, Substance use disorder, Addiction, Neuropsychiatry

## Abstract

Recent research with classic psychedelics suggests significant therapeutic potential, particularly for neuropsychiatric disorders. A mediating influence behind symptom resolution is thought to be the personal insight – at times, bordering on the mystical – one acquires during the acute phase of a psychedelic session. Indeed, current clinical trials have found strong correlations between the acute subjective effects (ASE) under the influence of psychedelics and their enduring therapeutic properties. However, with potential barriers to widespread clinical implementation, including the healthcare resource-intensive nature of psychedelic sessions and the exclusion of certain at-risk patient groups, there is an active search to determine whether ASE elimination can be accompanied by the retention of persisting therapeutic benefits of these class of compounds. Recognizing the aberrant underlying neural circuitry that characterizes a range of neuropsychiatric disorders, and that classic psychedelics promote neuroplastic changes that may correct abnormal circuitry, investigators are rushing to design and discover compounds with psychoplastogenic, but not hallucinogenic (i.e., ASE), therapeutic potential. These efforts have paved the discovery of ‘non-psychedelic/subjective psychedelics’, or compounds that lack hallucinogenic activity but with therapeutic efficacy in preclinical models. This review aims to distill the current evidence – both clinical and preclinical – surrounding the question: can the ASE of classic psychedelics be dissociated from their sustained therapeutic properties? Several plausible clinical scenarios are then proposed to offer clarity on and potentially answer this question.

## Introduction

The resurgence of classic psychedelic research after a decades-long hiatus is noteworthy. Early findings from research in the 1950s and 60s, although unmatched by the rigors of scientific conduct today, proved prescient in their conclusions about psychedelic therapeutic potential for neuropsychiatric disorders (Abuzzahab & Anderson 1971; Fuentes et al. 2019; Krebs & Johansen 2012; Rucker et al. 2016). The irony lies perhaps in the potential reasons for outlawing and thus decelerating research in psychedelic compounds through their insertion in the restrictive Schedule I category of the Controlled Substances Act, 1970 (Nutt et al. 2013): the same acute subjective effects (ASE) that were perceived as contributing to the 1960s counterculture movement are now considered instrumental, even if implicitly, in producing the enduring therapeutic outcomes across a spectrum of neuropsychiatric conditions (Bogenschutz et al. 2022; Carhart-Harris et al. 2016; Davis et al. 2021; Ehrmann et al. 2022; Gasser et al. 2015; Griffiths et al. 2016; Johnson et al. 2017; Lee & Shlain 1992; Mithoefer et al. 2016; Moreno et al. 2006; Palhano-Fontes et al. 2019; Schmid et al. 2021; van der Meer et al. 2023; Zeifman et al. 2019). ASE, as used in this review, refers to the initial minutes to hours following administration of a psychedelic compound that are characterized by profound alterations in perception, cognition, and mood.

A particular aspect of ASE that has garnered considerable attention is ‘mystical’ experiences occasioned by moderate to high doses of classic psychedelics (for psilocybin, moderate dose: 20 mg/70 kg; high dose: 30 mg/70 kg), with parallels drawn to ‘The Varieties of Religious Experience’ described by William James in 1902 (Garcia-Romeu et al. 2021; Griffiths et al. 2006; James & Ralph Ellison Collection (Library of Congress), 1902; Johnson et al. 2014; Yaden & Newberg 2022). Phenomenologically, a common core of features has been described in reference to mystical experiences that are independent of the interpretational framework through which such experiences are viewed (Stace & Bollingen Foundation Collection (Library of Congress), 1960). Although subsequent iterations have led to abbreviations of the original questionnaire, key features probed by the tool for the mystical experience remain similar: introvertive/extrovertive unity, spatial and temporal transcendence, positive mood, sacredness, objectivity (noetic quality), paradoxicality, and ineffability (Barrett & Griffiths 2018; Barrett et al. 2015; Johnson et al. 2019). Importantly, these experiences are viewed as mediating influences in positive therapeutic outcomes (Garcia-Romeu et al. 2014; McCulloch et al. 2022). A key double-blind clinical study of high-dose psilocybin (30 mg/70 kg) in healthy volunteers demonstrated the ability of such compounds to elicit experiences similar to spontaneously occurring mystical experiences as measured by an empirically derived, extensively studied scale (Griffiths et al. 2006). Follow-up studies at 14 months revealed enduring positive changes in the well-being of a majority of participants, with correlation and regression analyses implicating a central role of the mystical experience in such sustained positive outcomes (Griffiths et al. 2008).

In contrast, compelling recent studies in pharmacology and medicinal chemistry have cast doubt on the necessity of ASE for the sustained therapeutic properties of psychedelic compounds. By increasingly narrowing in on receptor/ligand pharmacology, investigators have designed reduced or non-hallucinogenic variants of psychedelic compounds that continue to activate the canonical 5-HT2A receptor (5-HT2AR) – widely considered the neurobiological target of classic psychedelics – and yet abolish the ASE while retaining therapeutic potential in preclinical animal models (Cameron et al. 2021; Dunlap et al. 2020; Kaplan et al. 2022). This approach aligns with the psychoplastogenicity model of classic psychedelic mechanism of action – one of many neurobiological theories currently being explored. Viewed this way, classic psychedelics are ‘psychoplastogens’ – capable of inducing measurable structural changes within the prefrontal cortex at the neuronal level within a short period of time following a single administration – and any elicited subjective experiences are “merely epiphenomena of underlying neurobiological mechanisms which convey the beneficial effects” (Ly et al. 2018; Olson 2018; Yaden & Griffiths 2021). Is it possible then to divorce the ASE – particularly the more profound alterations in consciousness induced by classic psychedelics – from the sustained therapeutic properties of these compounds?

The purpose of this review stems from recent debates about the necessity of ASE for sustained clinical benefits of classic psychedelics (Majic et al. 2015; Nautiyal & Yaden 2023; Olson 2021; Yaden & Griffiths 2021). Given the implications of this critical question on i) the integration of psychedelic compounds into clinical practice, ii) the pharmacologic design of similar or more effective compounds, and iii) a deeper mechanistic understanding of psychedelic action and, more generally, the contents of human consciousness, we present key recent findings that reinforce each or both sides of the debate. Rather than attempt to answer the question – which we suspect has considerable unexplored biologic nuance than a single review can capture – we describe the current state of the field drawing on recent preclinical and clinical data – the latter focusing on psilocybin as a representative classic psychedelic given that most recent clinical trials have studied this compound.

### Defining classic psychedelics and their acute subjective effects (ASE)

The ‘classic psychedelic’ rubric includes those compounds that elicit their ASE through agonism or partial agonism of the 5-HT2A receptor (psilocybin, lysergic acid diethylamide (LSD), mescaline, dimethyltryptamine (DMT) and related conformationally restricted analogs) and excludes empathogenic/entactogenic (MDMA) and dissociative (ketamine) agents that, while retaining psychoplastogenic properties, are characterized by sufficiently distinct receptor pharmacology (de la Torre et al. 2004; Kalant 2001; Madsen et al. 2019; Nichols 2016; Zanos et al. 2018). In essence, the categorization here is one driven by pharmacology (David E. Nichols, 2022). ASE, by definition, are subjective and thus exhibit inter-individual variation; however, given the consistent emergence of certain experiential patterns, instruments such as the mystical experience questionnaire (MEQ) and the altered states of consciousness questionnaire (ASC) have been developed that capture the core elements of the ASE. The ASC is a widely used psychometric measure of psychedelic-induced ASE that underwent revision to expand upon captured dimensions (Dittrich 1998; Studerus et al. 2010). The strengths of this revision (i.e., from the 5 Dimension (5D)-ASC to the 11D-ASC) include less ambiguous correlations with other measures, ease of interpretability, and a more granular account of ASE. Much like related predecessor scales derived from shared components in ‘mystical-type’ experiences independent of religious or cultural context, the ASC questionnaire encapsulates common core features in psychedelic-induced states of consciousness including altered perception (synesthesia and visual hallucinations at low and high doses, respectively), negative, anxiety-ridden experiences, and mystical experiences (Dittrich 1998; Hood 1975; Maclean et al. 2012; Pahnke et al. 1970; Pahnke & Richards 1966; Studerus et al. 2010). Similarly, the MEQ is a broadly adopted instrument that characterizes mystical experiences (over mere intensity of drug effects) and its psychometric fitness has been demonstrated in prospectively obtained experimental data (Barrett et al. 2015). Additional psychometric scales attempting to capture themes in ASE have also been used and described (e.g., challenging experience questionnaire, CEQ; emotional breakthrough inventory, EBI; hallucinogen rating scale, HRS) (Barrett et al. 2016; Riba et al. 2001; Roseman et al. 2019). Even within the class, however, there exists substantial variability in the duration of ASE from one compound to another (e.g., 15–30 min for DMT while 8 or more hours for LSD)(NIDA (2019)). As discussed later, this variability in ASE duration may have direct implications for the durability of therapeutic response (Nardou et al. 2023). What remains similar across individual compounds during this acute phase is the quality and the general impact of the effects on cognitive domains and affective measures (Holze et al. 2022; Ley et al. 2023). Physiologic effects (e.g., nausea and tachycardia) are also common with classic psychedelics, reflecting the preponderance of 5-HT2AR distribution outside of the central nervous system.

Lastly, for the purposes of this review, the scope of ‘therapeutic effects’ is restricted to those occurring in the context of neuropsychiatric disorders (affective disorders, substance use disorder (SUD), etc.) and not for other disorders for which psychedelic therapeutic potential has also been explored (chronic pain, cluster headache, etc.) (Schindler 2022; Schindler & Hendricks 2023; Schindler et al. 2022).

## The evidence from clinical studies

Relevant studies are listed in Table 1 along with the study design, agent used, sample size, clinical condition investigated, primary outcome, the means of ASE measurements, and the strength of association between ASE and primary outcomes.

**Table 1:** Studies favoring the necessity of ASE for therapeutic benefits

Clinical Study	Study Design	Neuropsychiatric Condition	n	Primary Outcome	Agent/Dosage	Measure of ASE	Strength of ASE/Primary Outcome (r)	Notes
(Goodwin et al. 2022)	Double-Blind, Dose-Finding, Parallel-Group, Randomized Control Trial	MDD	233	Δ in depression severity via total MADRS score	Psilocybin/25 mg, 10 mg, 1 mg	5D-ASC, EBI, PANAS (Positive and Negative Affect Schedule)	Correlation between changes in MADRS score and 3 dimensions of 5D-ASC [Oceanic Boundlessness (r = -0.508, 25 mg; r = -0.485, 10 mg; r = -0.477, 1 mg), Visual Restructuralization (r = -0.516, 25 mg; r = -0.431, 10 mg; r = -0.410, 1 mg), Auditory Alterations (r = -0.358, 1 mg)] and EBI total score (r = -0.614, 25 mg; r = -0.363, 10 mg; r = -0.424, 1 mg)	Improvement in depressive symptoms (from baseline to week 3) correlated with higher scores on 3 dimensions of 5D-ASC and EBI total score(Goodwin 2022); See Goodwin et al. (Goodwin et al. 2023) for results on additional secondary outcomes
(Carhart-Harris et al. 2016)	Open-label Feasibility Trial	MDD	19	Δ in depressive symptom (self-reported) severity (QIDS-SR-16), patient-rated subjective intensity of psilocybin	Psilocybin/10 mg, 25 mg	11D-ASC	Components of the 11D-ASC [USB (experience of unity, spiritual experience, blissful state) and insight] correlated with changes in QIDS-SR16 scores with 25 mg psilocybin: r = -0.49 (USB), r = -0.57 (insight)	Significant relationships exist between 11D-ASC (inter-related factors and individual components) and changes in QIDS-SR-16 at 5 weeks in high-dose group (Carhart-Harris et al. 2018a, b); Significant relationship between 'peak experience' and personality changes also found (Erritzoe et al. 2018)
(Davis et al. 2021)	Randomized Controlled Trial	MDD	24	Δ in depression severity (GRID-HAMD scores)	Psilocybin/20, 30 mg/70 kg	MEQ30, CEQ, Peak ratings	MEQ30 (r = -0.41) correlated with GRID-HAMD scores	Intensity and import of ASE (e.g., degree of personal meaning, psychological insight, and spiritual significance) had moderate-to-strong correlations with decrease in depression scores at 4 weeks. Although correlation exists between highest scores on MEQ and decrease in depression, a complete mystical experience not necessary for therapeutic benefit. See eTables 4–6 in Supplement 2 for more information
(Ross et al. 2016)	Double-Blind, Cross-Over, Randomized Control Trial	MDD and Anxiety (in terminally ill cancer patients)	29	Δ in depression and anxiety severity (HADS, HAD-A, HADS D, HADS T, BDI, STAI S, STAI T)	Psilocybin/0.3 mg/kg	MEQ30, HRS	MEQ30 correlated with 5 of 6 primary outcomes: HADS T (Spearman r = 0.49), HADS A (r = 0.46), HADS D (r = 0.35), BDI (r = 0.48), STAI S (r = 0.42), STAI T (r = 0.40)	Total MEQ scores at the end of dose 1 correlated with change scores (baseline to 6 weeks) on five of six primary outcome measures: HADS T; HADS A; BDI; STAI S; STAI T. Total MEQ scores mediated therapeutic benefit. For a similar study, see Grob et al. (Grob et al. 2011)
(Griffiths et al. 2016)	Double-Blind, Cross-Over, Randomized Control Trial	MDD and Anxiety (in terminally ill cancer patients)	51	Response and Remission as determined by scores on GRID-HAM-D-17, HAM-A (assessed with SIGH-A)	Psilocybin/1, 3, 22, 30 mg/70 kg	HRS, 5D-ASC, Mysticism Scale, SOCQ (includes MEQ30)	MEQ30 correlated with primary outcome: GRID-HAMD (r = -0.41) and HAM-A (r = -0.59)	MEQ30 scores correlated significantly with numerous outcome measures (GRID-HAMD, HADS Depression, HADS Anxiety, HAM-A) 5 weeks after the psilocybin session. MEQ30 scores significant mediators of the effect of psilocybin dose on some outcome measures (HADS Anxiety, HADS Depression, HADS total, and HAM-A). For a similar study, see Grob et al. (Grob et al. 2011)
(Bogenschutz et al. 2015)	Proof-of-Concept, Open-Label, Pilot, Single-Group, Within-Subjects Trial	AUD	10	Drinking behavior (TLFB, Drinking Days, Short Inventory of Problems, multiple psychological assessments)	Psilocybin/0.3, 0.4 mg/kg	HRS, 5D-ASC, SOCQ, Total MEQ	HRS, Total MEQ, and G-ASC all correlated with outcome measures: PDD [r = -0.844 (HRS), -0.885 (MEQ), -0.838 (G-ASC)], PHDD [r = -0.763 (HRS), -0.852 (MEQ), -0.893 (G-ASC)], PACS [r = -0.823 (HRS), -0.810 (MEQ)], AASE [r = 0.753 (HRS), 0.762 (MEQ)]	Large correlations observed between ASE (three summary measures: HRS, G-ASC, and MEQ) and short-term clinical outcomes (Δ PDD, PHDD, PACS scores, AASE confidence)
(Bogenschutz et al. 2022)	Double-Blind, Randomized Controlled Trial	AUD	48	Δ change in percentage of heavy drinking days (PHDD) (assessed by TLFB)	Psilocybin/20, 30, 40 mg/70 kg	SOCQ, MEQ	See notes	Although higher average scores for 'intensity of experience' in the treatment (psilocybin) vs placebo (diphenhydramine) arm, study did not explicitly report an association between ASE (intensity of experience as measured by MEQ) and treatment response (lower PHDD)
(Johnson et al. 2014)	Open-Label Pilot Trial	Tobacco Use Disorder	15	Smoking Cessation (with various measures)	Psilocybin/20–30 mg/70 kg	SOCQ, Mysticism Scale, Visual Effects Questionnaire	Mystical experience score of SOCQ correlated with Change in Questionnaire on Smoking Urges (QSU) (r = -0.65), MEQ30 correlated with Δ in urinary cotinine levels (r = -0.55)	Δ in urinary cotinine levels (a biological measure of smoking abstinence) significantly associated with average MEQ, SOCQ, and *Questionnaire on Smoking Urges* (QSU) craving scores. Personal meaning attributed to dosing sessions significantly correlated with Δ scores in a range of measures. See also Johnson et al. (Johnson et al. 2017)
(Aaronson et al. 2023)	Open-Label Pilot Trial	MDD in Bipolar II disorder	15	Δ in depression severity via total MADRS score	Psilocybin/25 mg	5D-ASC	4 of 5 dimensions of 5D-ASC correlated with MADRS score: OB (r = -0.66), AED (r = -0.55), VR (r = -0.79), RV (r = -0.59); Global 5D-ASC score correlated with MADRS score at 12 weeks (r = -0.69)	4 of 5 dimensions of 5D-ASC (OB, AED, VR, RV) significantly correlated with MADRS end point at 3 weeks. Global 5D-ASC score significantly correlated with MADRS scores at 12 weeks

The bulk of evidence favoring the necessity of ASE for enduring therapeutic benefits consists of associations between psychometric scores (ASC, MEQ, etc.) and improved clinical outcomes. As others have pointed out, however, these do not amount to causation (Olson 2021). Nevertheless, such associations are compelling enough that the enhancement of ASE are operational principles underlying clinical trials, i.e., study designs include efforts to facilitate the production of ASE through careful modification of set/setting (O'Donnell et al. 2022). The importance of set (the mental well-being of an individual) and setting (physical environment of an individual) cannot be overstated (Carhart-Harris et al. 2018a, b). For the latter, modern clinical trials aspire towards creating naturalistic settings with subjects undergoing psilocybin sessions in aesthetically pleasing environments (e.g., living room-like settings with comfortable furniture, décor, art, plants, personal memorabilia, etc.) while frequently lying supine with eyeshades on and listening to pre-selected music delivered through headphones (Kaelen et al. 2018; Strickland et al. 2021). Additionally, extensive psychotherapeutic sessions flank the focal drug session to maximize therapeutic benefits. In fact, the term ‘psychedelic-assisted therapy’ stems from a combination of such psychotherapy sessions and psychedelics, in which the latter is viewed as an adjunct to the former. The presence of study monitors may also serve to accentuate ASE by providing a perceived safe environment for the patient to experience an altered state of consciousness. This is achieved through guidance and grounding exercises – typically by trained psychologists who sit for the duration of the ASE – for the patient during the acute changes of a psychedelic experience. This undoubtedly, however, contributes to the high cost of implementing this model, particularly on a larger scale. Strict exclusion criteria for patients with relevant neuropsychiatric history exemplifies the adherence to ‘set’ in participant selection (Johnson et al. 2008; O'Donnell et al. 2022). Abiding by such standards of set/setting emphasizes the importance ascribed to nondrug parameters in contemporary psychedelic clinical research. Notwithstanding their obvious role as safety valves against psychologically challenging experiences triggered by classic psychedelics, there is concern that their influence on therapy may obscure true effect sizes of the pharmacologic agent. The dissection of set/setting from the direct therapeutic properties of classic psychedelics is a subject of active discussion and a resolution remains to be seen (Aday et al. 2022; Hartogsohn 2016).

### Depression

Carhart-Harris et al. conducted an open-label feasibility study for protocol optimization and preliminary assessment of treatment efficacy for psilocybin administration (fixed dose, 10 mg and 25 mg) plus psychological support in patients with unipolar treatment-resistant depression (Carhart-Harris et al. 2016). Multiple time-points (baseline, 1/2/3/5 weeks, 3 months) were included to measure the outcome of interest (change in the severity of depressive symptoms as measured by the 16-item Quick Inventory of Depressive Symptomatology (QIDS score)). ASE were measured retrospectively (6–7 h after dosing with reference to the period when effects were most intense) with the 11D (11-dimension)-ASC. Subsequent sub-analyses of the original cohort revealed ASE to be good predictors of clinical outcomes (Roseman et al. 2017). Specifically, when testing the hypothesis that the quality of ASE would predict treatment efficacy and that, by extension, certain components of the 11D-ASC – *oceanic boundlessness* (OBN), a partial surrogate for mystical-type experiences, and *dread of ego dissolution* (DED), reflective of anxiety – would predict clinical outcomes at 5 weeks, it was found that high OBN and low DED scores predicted improvements in depressive symptoms. Importantly, this predictive association was specific for the mystical/peak experiential components and not the perceptual disturbances that compose the ASE. A 6-month follow-up of the original trial lumped together specific interrelated factors of the 11D-ASC (*experience of unity*, *spiritual experience*, and *blissful state*; *USB*) and assessed their predictive potential along with that of acute *insight* – also a measured factor in 11D-ASC – for improved clinical outcomes (Carhart-Harris et al. 2018a, b). Again, significant relationships were found between mean scores of USB and insight during the high-dose psilocybin session and changes in QIDS-SR16 scores at 5 weeks. Interestingly, subsequent exploratory correlation analyses assessing the relationship between peak experience and personality changes – as measured by NEO-PI-R trait scores – found the degree of *insightfulness* during the high-dose psilocybin session to be significantly associated with a reduction and increase in scores of *neuroticism* and *extraversion*, respectively, at 3-month follow-up (Erritzoe et al. 2018). Similarly, *spiritual experience* was significantly positively correlated with increased *extraversion* from baseline to follow-up. It is worth acknowledging, however, that a subsequent expanded phase 2 trial led by Carhart-Harris et al. investigating psilocybin treatment efficacy (fixed dose, 1 mg and 25 mg) against escitalopram found no correlation between ASE (as measured by EBI scores) and the primary clinical outcome (change from baseline in QIDS-SR-16 scores) (Carhart-Harris et al. 2021). A key difference between the two aforementioned studies was study design (i.e., the presence of blinding in the phase 2 trial versus the open-label study), which may have factored in to the loss of association. Results from the largest phase 2 multicenter, double-blind randomized trial evaluating a single-dose of psilocybin (fixed dose, 1/10/25 mg) for treatment-resistant depression have shown efficacy for a 25-mg dose (Goodwin et al. 2022, 2023). Although exploratory analyses investigating the correlation of ASE (as measured by 5D-ASC and EBI) with the primary endpoint (change in total *Montgomery-Asberg Depression rating Scale,* MADRS, score from baseline to week 3 and 12) have yet to be published, preliminary results suggest a dose–response relationship supporting the role of ASE in mediating therapeutic outcomes (Goodwin 2022). More recently, another large phase 2 trial of a single 25 mg dose of psilocybin demonstrated efficacy in patients with MDD of a moderate or greater severity (Raison et al. 2023). The psilocybin arm (n: 51), compared to the active placebo (niacin; 100 mg) arm (n: 53), was significantly associated with a reduction in MADRS score from baseline to day 43 (primary outcome) and day 8 (secondary outcome). Unfortunately, no mention of an ASE measure was made in the original publication, and thus it is unclear an association between ASE and outcomes was found. However, results of this trial are generally supportive of, and add to the mounting evidence for, a therapeutic role of psilocybin in mood disorders.

Similarly, Davis et al. investigated the value of psilocybin-assisted therapy in MDD in a randomized clinical trial employing two psilocybin sessions (weight-adjusted dose, 20 mg/70 kg, 30 mg/70 kg; sessions separated by a week) in the context of extensive supportive psychotherapy (Davis et al. 2021). The primary outcome was change in depression severity (assessed with GRID- *Hamilton Rating Scale for Depression,* GRID-HAMD scores) at weeks 1 and 4 following the second dosing session. A range of measures were included to evaluate the intensity and importance of ASE: participants rated the degree of *personal meaning*, *psychological insight*, and *spiritual significance* of the ASE under the influence of psilocybin, and all three ratings had a significant and moderate-to-strong correlation with decreases in depression scores at week 4. Other facets of ASE explored were the extent of mystical (MEQ) and challenging experiences (CEQ). Intriguingly, despite a correlation between the highest scores on the MEQ and decrease in depression, the occurrence of a *complete mystical experience* (≥ 60% of maximum score on all 4 factors of the MEQ) did not appear necessary for therapeutic benefit. No significant correlation was found between scores on the CEQ and changes in depression.

In the first modern clinical trial of psilocybin in bipolar type II disorder (BDII), Aaronson et al. explored the safety and efficacy of a single administration of 25 mg psilocybin in 15 patients with BDII (Aaronson et al. 2023). Assessing change in MADRS total score from baseline to 3 weeks’ follow-up (efficacy endpoint), the investigators found lower scores in all participants (mean reduction of 76%), with 12 and 11 participants meeting response and remission criterion, respectively. Of relevance, an exploratory endpoint to assess the correlation of ASE (measured with the 5D-ASC) and antidepressant efficacy found 4 of 5 dimensions of the 5D-ASC to significantly correlate with the MADRS 3-week end point. Furthermore, a global 5D-ASC score (summation across all subscales as a measure of overall intensity of ASE) was also found to significantly correlate with MADRS scores at week 12, suggesting the predictive potential of ASE for longer-term antidepressant effects.

Several trials have evaluated the efficacy of psilocybin in alleviating psychological distress (depressed mood and anxiety) associated with advanced-stage cancer. In a randomized double-blind, cross-over study design, Griffiths et al. demonstrated that a single high dose (weight-adjusted dose, 22 mg/70 kg) of psilocybin, in tandem with psychological support, significantly decreased clinician- and self-rated measures of depressive (GRID-HAMD17, *Beck Depression Inventory,* BDI, and *Hospital Anxiety and Depression Scale,* HADS Depression) and anxiety (*Hamilton Anxiety Rating Scale,* HAM-A, *State-Trait Anxiety Inventory,* STAI-Trait Anxiety) symptoms in patients with a life-threatening cancer diagnosis (Griffiths et al. 2016). Of relevance, scores on the MEQ30 (one of four ASE measures) after controlling for participant-rated intensity of drug effect (‘intensity’ item on HRS completed 7 h after drug administration) correlated significantly with numerous outcome measures (GRID-HAMD, HADS Depression, HADS Anxiety, HAM-A) 5 weeks after the psilocybin session. The correction for participant-rated drug intensity was performed to test for associations between specific components of ASE (mystical experience in this case) and primary outcomes. Additionally, a mediation analysis found MEQ30 scores as significant mediators of the effect of psilocybin dose on some of these outcome measures (HADS Anxiety, HADS Depression, HADS Total, and HAM-A), implicating thus the predictive potential of psilocybin-induced mystical experiences for positive therapeutic responses. The findings from this study were consistent with, and an extension of, a prior safety and feasibility trial conducted by Grob et al. where even a lower dose of psilocybin (weight-adjusted, 0.2 mg/kg) administered in terminally-ill cancer patients demonstrated a trend in favor of psilocybin for depression (BDI, but not POMS, scores displayed significant sustained improvements at a 6-month follow-up point) and anxiety (STAI trait, but not state, subscale displayed sustained reductions reaching significance at the 1- and 3-month follow-up point) (Grob et al. 2011). However, despite measurements of ASE (via the 5D-ASC), correlations of ASE with therapeutic outcomes were not performed owing to the lower dose and thus a lower expectation of profound transcendental states – associated with improved outcomes – typically elicited by higher doses. Ross et al. further reinforced the therapeutic value of psilocybin in cancer-associated psychological distress by carrying out a randomized, double-blind, crossover trial using a single psilocybin dosing session (weight-adjusted dose, 0.3 mg/kg) (Ross et al. 2016). The primary outcomes, prior to crossover, were assessment of depression and anxiety at various timepoints. After controlling for participant-rated drug intensity (item 98 on HRS scale), significant effects of total MEQ scores on changes in five of six primary outcome measures (HADS T, HADS A, BDI, STAI S, STAI T) were found at six weeks following the psilocybin dose. Here too the intensity of the subjective experience – specifically the mystical experience component – was found to significantly mediate the therapeutic benefit (anxiolytic, antidepressive) in the medium term (six-weeks).

A number of clinical studies in depression have also recently emerged that did not find associations between ASE and therapeutic benefits. von Rotz et al. performed a placebo-controlled trial of a single moderate dose of psilocybin in 52 MDD patients (von Rotz et al. 2023). Primary efficacy endpoints were defined as changes in symptom severity as measured by MADRS and BDI. The ASC questionnaire was used to quantify ASE, with scale scores reported as a percentage of the maximum scale value. A global intensity score was then calculated as the mean of all main dimensions (excluding vigilance reduction) with higher scores representing greater degrees of ASE. Although the psilocybin arm showed significant improvements in symptom severity above those found in the placebo arm, and ASE were, unsurprisingly, significantly higher in the psilocybin arm, the overall global intensity scores did not significantly differ between responders and non-responders (in either arm) at 2-weeks post treatment. Similarly, Sloshower et al., although originally investigating an electrophysiological biomarker of psilocybin-induced neuroplasticity, were able to report results from an exploratory analysis for their placebo-controlled, fixed-order trial of psilocybin (0.3 mg/kg) for MDD. They too did not find significant correlations between ASE (as measured by MEQ-30 and CEQ ratings) and changes in depression scores. Notably, they also did not find significant differences in the primary outcomes (change in GRID-HAM-D-17 scores two weeks after each dosing session) although they saliently highlight possible reasons for this observation (underpowered sample size, carryover effects from the placebo arm, expectancy effects, and others).

### Alcohol and substance use disorder

In a proof-of-concept study, Bogenschutz et al. used a single-group, within-subjects design to administer two open-label psilocybin sessions (weight-adjusted dose, 0.3 and 0.4 mg/kg) to ten participants with a DSM-IV diagnosis of alcohol use disorder (AUD). Consistent with results from a meta-analysis evaluating early clinical trials (1960/70 s) of LSD for AUD, this study provided additional evidence for a quantitatively robust response of psilocybin for abstinence in AUD, a response that was maintained at follow-up to 36 weeks (Bogenschutz et al. 2015; Krebs & Johansen 2012). Using three summary measures of ASE (HRS, G-ASC, and MEQ), large correlations were observed between ASE and short-term clinical outcomes (changes in *Percentage Drinking Days,* PDD, *Percentage Heavy Drinking Days,* PHDD, *Penn Alcohol Craving Scale,* PACS scores, *Alcohol Abstinence Self-Efficacy Scale,* AASE). It is worth noting, however, that a decision to increase to a higher dose (from 0.3 to 0.4 mg/kg) in the second session rested on selection criteria that included total MEQ scores, with patients lacking a complete mystical experience in the first session given the higher dose in the second session. This, in turn, may have contributed to the relatively strong correlations seen between ASE measures and outcomes. Importantly, this study set the stage for a larger phase 2 clinical trial also conducted by Bogenschutz et al., the results of which were, again, supportive of a role of psilocybin (weight-adjusted dose, 20/30/40 mg/kg) for the treatment of AUD (Bogenschutz et al. 2022). Specifically, in combination with psychotherapy, psilocybin produced substantial decreases in PHDD (-32.4%; primary outcome of study). Although the association between ASE and treatment response was not explicitly reported, the investigators reported higher average MEQ scores in the treatment (psilocybin) versus placebo (diphenhydramine) arm.

Johnson et al. carried out an open-label pilot study to assess the safety and feasibility of psilocybin-facilitated smoking cessation treatment (Johnson et al. 2014). Compared to abstinence rates with conventional pharmacotherapies for smoking cessation (26%, buproprion; 38%, varenicline), 80% of participants administered psilocybin sessions (weight-adjusted dose, 20 and 30 mg/70 kg) showed seven-day point prevalence abstinence at 6-month follow-up (Anthenelli et al. 2016). Despite the obvious caveat of a small sample size (*n* = 15) and open-label design, the study highlights the feasibility of conducting larger randomized trials. Of relevance, a biological measure of smoking abstinence (change in urinary cotinine levels) was found to significantly associate with average MEQ, *States of Consciousness Questionnaire* (SOCQ), and *Questionnaire on Smoking Urges* (QSU) craving scores (obtained at the conclusion of psilocybin session) from study initiation to long-term follow-up (Garcia-Romeu et al. 2014; Johnson et al. 2017). Personal meaning attributed to the sessions also significantly correlated with change in scores of breath carbon monoxide (CO), urine cotinine, *Timeline Followback* (TLFB) daily smoking, *Smoking Abstinence Self-Efficacy* (SASE) confidence to abstain, and SASE temptation to smoke (Garcia-Romeu et al. 2014).

### Eating disorders

Peck et al. evaluated the safety, tolerability, and exploratory efficacy of a single administration of 25 mg psilocybin accompanied by psychological support in 10 patients with anorexia nervosa (AN)(Peck et al. 2023). Importantly, although a correlation of ASE (measured with 5D-ASC) and outcomes was not reported, this open-label trial demonstrated safety and feasibility of this treatment paradigm for a deadly illness with no approved pharmacological interventions. Significant reductions at 1-month follow-up were noted for changes in psychopathology (secondary outcomes) across a range of domains: weight concerns (1- and 3-month), trait body image anxiety, trait anxiety, preoccupations and rituals surrounding food, eating and shape, and functional impairment related to eating disorder. However, most of these results were highly variable among the ten participants and the limited sample size further restricts broad conclusions.

## The evidence from preclinical studies

The psychoplastogenic theory of psychedelic mechanism of action (i.e., the neuropsychiatric therapeutic mechanism of action) suggests that psychoplastogens are small molecules that “produce a measurable change in plasticity (e.g., changes in neurite growth, dendritic spine density, synapse number, intrinsic excitability, etc.) within a short period of time (typically 24–72 h) following a single administration” (Olson 2018). A further defining characteristic is the long-lasting changes in mood and behavior that outlast the presence of the drug, presumably due to neuroplasticity-enabled reshaping of aberrant neural circuitry. In keeping with the psychoplastogenic model of psychedelic mechanism of action – a model shaped by elegant mechanistic studies of ketamine’s antidepressant-like effects (Li et al. 2010; Moda-Sava et al. 2019) and later molded by work with classic psychedelics (Jones et al. 2009; Ly et al. 2018; Marinova et al. 2017; Shao et al. 2021) – efforts are underway to recapitulate neuroplasticity with compounds structurally similar to classic psychedelics but lacking hallucinogenic potential. Despite the imprecision of the term ‘hallucinogenic potential’ (frank hallucinations occur primarily with higher doses of psychedelics), the profound alterations in consciousness that accompany the acute effects of classic psychedelics in humans are seen as disruptive to practical considerations of psychedelic clinical implementation (patient throughput/access, healthcare costs, etc.) (Muthukumaraswamy et al. 2022; Olson 2021). Thus, from the standpoint of clinical implementation, decoupling ASE from the persistent therapeutic benefits of classic psychedelics has considerable practical appeal. Unlike the evidence favoring the necessity of ASE, most work in this section (Table 2) derives from animal studies wherein psychoplastogenicity is more easily measured. Additionally, where ASE is to be measured, the head-twitch response (HTR) serves as a reliable, although arguably non-specific, behavioral surrogate of psychedelic potential in rodent models (Halberstadt et al. 2020; Hanks & Gonzalez-Maeso 2013).

**Table 2:** Studies favoring the decoupling of ASE and therapeutic benefits

Animal Studies	Agent	Structural Derivative	Animal(s)	Measure of Therapeutic Potential	Measure of ASE Potential	Notes
(Cameron et al. 2021) and (Lu et al. 2021)	Tabernanthalog	Ibogaine	Mice, zebrafish	Alcohol and heroin-seeking behavior (two-bottle choice experiment and heroin self-administration, respectively); Behavioral Despair (FST); Anxiety (elevated plus maze), Sensory Processing (whisker-dependent Texture Discrimination Task), Cognitive Flexibility (four-choice odor discrimination and reversal task)	HTR, behavioral studies in zebrafish	Decreased ethanol binge-drinking and diminished heroin-seeking behavior (during self-administration, before extinction and cued reinstatement) with lasting impact. Decreased immobility in FST. See (Lu et al. 2021) for rescue of behavioral deficits (anxiety, etc.) and assessment of dendritogenesis (a purported mechanism underlying therapeutic response)
(Kaplan et al. 2022)	(R)-69 and (R)-70	LSD	Mice	Behavioral Despair (FST, TST; Learned Helplessness Mice Model, Sucrose preference)	HTR, Prepulse Inhibition Test	VMAT2 heterozygous mice used to amplify anhedonic behavior (i.e., enhanced immobility). Leaned helplessness model consisted of foot-shock (FS) and non-FS assignments. Sucrose preference in FS mice was higher and TST immobility times lower after (R)-70 administration. Study originally identified 17 agents, two of which ((R)-69 and (R)-70) were tested in animal models
(Dong et al. 2021)	AAZ-A-154	DMT	Mice	Behavioral Despair (FST), Sucrose preference	HTR	Decreased immobility in FST. Sucrose preference in VMAT2-Het mice rose to WT control levels after AAZ-A-154 administration. Similar ligand score as TBG. In vivo, AAZ-A-154 may be more potent than TBG while producing more sustained antidepressant effects
(Cunningham et al. 2023)	Ariadne	Mescaline	Mice	Anxiety (Open-Field Test (OFT)), Novelty-suppressed Feeding Test	HTR	Anxiogenic effects in the OFT but lasting anxiolytic effects on NSFT. Therapeutic effects also evident in mice models of Parkinson's disease (relief of motor deficits)
(Lewis et al. 2023)	2-Br-LSD	LSD	Mice	Behavioral Despair (FST), Anxiety (OFT)	HTR	Decreased immobility in FST. Increased exploration of stressogenic environments as doses with no locomotor activity in female (not male) mice
(Dunlap et al. 2020)	5-MeO-isoDMT (and others)	DMT	Mice, zebrafish	-	HTR	Assessed psychoplastogenicity (a purported mechanism underlying therapeutic response) but not therapeutic effects
(Cao et al. 2022)	IHCH-7079, IHCH-7086	Many (serotonin, psilocin, atypical antipsychotics)	Mice	Behavioral Despair (FST, TST)	HTR	Antidepressive properties (reduced immobility in FST/TST) also evident in corticosterone-induced depression in mice
Clinical Studies in MDD						
Clinical Studies	Agent/Dosage	Study Design	n	Primary Outcome	Measure of ASE	Notes
(von Rotz et al. 2023)	Psilocybin	Double-blind, placebo-controlled, randomized control trial	52	Δ in depression severity (via MADRS and BDI scores)	ASC	No correlation between scores and reduction in depressive symptoms. No significant differences in scores between responders and non-responders in psilocybin or placebo condition
(Sloshower et al. 2023)	Psilocybin	Exploratory, placebo-controlled, fixed-order trial	34	Δ in depression severity (via GRID-HAM-D-17)	MEQ, CEQ	No significant correlations between MEQ and CEQ scores and changes in depression scores after psilocybin dosing

Structure–activity relationship (SAR) studies and the redesigning of traditional psychedelics has provided numerous candidate molecules that demonstrate the desired phenotype: lacking hallucinogenic properties but retaining therapeutic value. Dunlap et al. explored this strategy by identifying key features of the psychoplastogen pharmacophore (dimethyltryptamine, DMT) and engineering its derivatives (‘isoDMT’) capable of dendritogenesis, while lacking HTR responses in mice (Dunlap et al. 2020). Specifically, SAR studies identified the pharmacophore as a modifiable aromatic ring separated from a basic nitrogen by a short linker (Glennon et al. 1984). Furthermore, substitution at the 4-position of such derivatives (e.g., 5-MeO-isoDMT) eliminated hallucinogenic potential while retaining the neuroplastic properties – through binding at 5-HT2AR – at levels comparable to that of ketamine (Chang-Fong et al. 2002). Overall, they demonstrated the potential for modifying existing psychoplastogens with a DMT core structural feature, and then screening subsequent derivatives for efficacy, safety profiles, and hallucinogenic potentials. This approach has been utilized for numerous other established psychedelics and has led to the identification of many promising molecules with therapeutic potential, as described in the studies below.

In similar structure-based design experiments, Cao et al. recognized that ß-arrestin2 activity is important for the antidepressive properties of 5-HT2AR agonists but insufficient for hallucinogenic effects – the latter requiring high transduction efficiency at both G-protein mediated signaling and ß-arrestin recruitment (Cao et al. 2022). This recognition was triggered by the finding that serotonin and psilocin adopt two different positions at the 5-HT2AR that differentially affect function, one that is also modulated by certain lipids (monoolein). What followed was the development of ß-arrestin-biased ligands (‘IHCH-7079’ and ‘IHCH-7086’) without detectable Gq activity. Upon testing for hallucinogenic activity, IHCH-7079 and IHCH-7086 failed to produce any HTR and were also found to abolish LSD-induced HTR. In vivo studies of antidepressant potential, as measured by the FST and tail suspension test (TST), revealed significantly attenuated acute-restraint stress-induced immobility with the two ligands, an effect that was eliminated by a selective 5-HT2AR antagonist (MDL100907). These antidepressant properties were reinforced in mouse models of corticosterone-induced depression (i.e., enhanced immobility in FST and TST in mice subjected to 21 days of corticosterone treatment) where, like LSD, the two ligands reduced immobility. In sum, ß-arrestin-biased ligands with minimal Gq activity at 5-HT2AR could offer antidepressant properties without hallucinogenic potential.

Kaplan et al. employed an equally impressive drug-discovery methodology where compilation of a virtual library consisting of 75 million tetrahydropyridines – a class of six-membered nitrogen heterocycles present in LSD and a range of non-psychedelic compounds – ultimately furnished molecules with selectivity for 5-HT2R subtypes, including 5-HT2AR (Kaplan et al. 2022). After confirmation of the predicted structure of receptor-ligand complex with cryo-electron microscopy, these molecules (‘(R)-69’ and ‘(R)-70’) were validated in mice behavioral studies of anhedonia and psychedelic activity. The latter was determined to be negligible when both the HTR and prepulse inhibition (PPI) tests displayed near-absent responses with (R)-69 and (R)-70 relative to LSD. Therapeutically, the molecules exhibited antidepressive and anxiolytic features across a range of mouse models of depressive-like behavior. In the FST and TST, use of vesicular monoamine transporter 2 (VMAT2) heterozygous mice served to amplify behavioral despair above that of wild-type (WT) treated mice (i.e., enhanced immobility). Notably, administration of (R)-69 and (R)-70 reduced immobility times in the VMAT heterozygous mice to that of wild-type treated mice, a reduction that remained 24 h after administration suggesting antidepressive properties of the molecules in mice genetic models. Similarly, mice assigned to foot-shock (FS) and non-FS (NFS) conditions in a learned helplessness model of anhedonic- and anxiety-like behavior exhibited improvements of behavioral deficits with administration of (R)-70. Specifically, sucrose preference in FS mice administered (R)-70 was higher and immobility times in the TST lower than those of vehicle controls. Importantly, these improvements persisted over days. Changes in anxiety-like behavior were less robust. Although in the elevated zero maze test vehicle-treated FS mice spent less time in open areas compared with (R)-70-treated FS mice, escape testing (number of escapes and latency to escape) did not reveal much difference with (R)-70 treatment, implying that, at least at the doses used, anxiolytic behavior was less clear.

Other analogues have similarly shown promise for structural plasticity and positive therapeutic effects without ASE, including an LSD analogue, ‘2-Br-LSD’ (Lewis et al. 2023). Valles et al. found that 2-Br-LSD increased structural plasticity in rat cortical neurons and was able to significantly reduce anxiety- and depression-like behaviors following chronic-stress while simultaneously not inducing an HTR over a 100-fold range of doses. Aminergic G Protein-Coupled Receptor (GPCR) screening (using a G protein dissociation BRET-based assay platform optimized for 33 human GPCRs) found that while LSD was a near full agonist for 5-HT2AR and 5-HT2BR (a surprising finding given that LSD is typically regarded as a partial agonist as measured in functional studies), 2-Br-LSD acted as a partial agonist at 5-HT2AR and an antagonist for 5-HT2BR (Kurrasch-Orbaugh et al. 2003; Rickli et al. 2016). These properties of 2-Br-LSD are consistent with observations in prior studies (Harvey et al. 1982; Romano et al. 2000). Although evidence is yet to be found, 5-HT2BR antagonism may, in theory, decrease the potential risk of cardiac valvulopathy implicated in the chronic (not single or infrequent repeated) use of LSD and other psychedelics. 2-Br-LSD also showed reduced activation of additional GPCRs compared to LSD which could also limit other off-target effects. Although hallucinogenic psychedelics like LSD and psilocybin cause rapid and lasting structural plasticity with a single dose, they show rapid behavioral tolerance and downregulation of 5-HT2AR expression via ß-arrestin signaling. 2-Br-LSD, on the other hand, showed weak ß-arrestin recruitment and did not show signs of tolerance or 5-HT2AR downregulation.

Focusing on the mescaline lineage-derived, non-hallucinogenic psychedelic analogue, ‘Ariadne’ (2-amino-1-(2,5,dimethoxy-4-methylphenyl)-butane), Cunningham et al. revived investigation of its pharmacologic profile both in vitro and in vivo (Cunningham et al. 2023). The compound originally gained popularity in the 1970s under the auspices of Bristol-Myers when it was touted to hold considerable clinical therapeutic potential based on animal and limited human studies (Richard A. Partyka, 1977; Standridge et al. 1980). Unfortunately, the clinical data were never made publicly available and drug development was halted. Following synthesis, Ariadne was screened against a range of molecular targets to determine receptor specificity and, in keeping with a serotonergic mechanism of action, 5-HT2A and 5-HT2B emerged as top hits. This was further confirmed by a functional assay that demonstrated robust agonist activity at 5-HT2A/2B/2C for racemic and R-enantiomer of Ariadne. Importantly, agonist activity at 5-HT2A was substantially more potent than at 5-HT2B/2C. Ariadne’s lack of hallucinogenic activity was demonstrated by a significantly suppressed HTR in mice. To elucidate underlying signaling pathways responsible for non-hallucinogenicity, two primary effector assays using BRET (Gq disassociation and β-arrestin2 recruitment assays) were used. Interestingly, in comparison with a hallucinogenic counterpart (2,5-Dimethoxy-4-methylamphetamine, DOM), Ariadne did not reveal a bias or preference between Gq and β-arrestin2 signaling pathways but instead there was a modest and consistent loss of potency and efficacy across all surveyed signaling pathways. Mouse behavioral studies displayed acute anxiogenic effects in the open-field test and lasting anxiolytic effects (7 days post drug administration) based on the novelty-suppressed feeding test. Therapeutic effects were more pronounced in mouse models of Parkinson’s disease, where Ariadne was shown to relieve motor deficits typically associated with the disorder. Specifically, the investigators employed an auxilin knockout (Aux KO) mouse model of PD, a model previously described to share classic features of PD (Vidyadhara et al. 2023). 9–12 months old Aux KO mice treated with Ariadne (10 mg/kg) demonstrated significant improvement in various motor behavior tests (balance beam and hind limb clasping tests) when compared to wild-type mice.

Other screening tools have also been developed to monitor specific signal cascades downstream of 5-HT2AR activation. Understanding different conformational states in the 5-HT receptors may help answer why certain serotonergic ligands are hallucinogenic and others are not. Not only will this enable better drug design, but will enhance our understanding of how ligand structure may influence receptor conformation, downstream signaling, and ultimately distinct functional and behavioral consequences. This was achieved by the genetically encoded fluorescent sensor, “Psychlight”, the first 5-HT sensor specifically based on the 5-HT2AR, providing measures of conformational changes as opposed to ligand/receptor interactions or indirect secondary signaling (Dong et al. 2021). By directly measuring 5-HT2AR conformational changes, it allows for detection of a wide range of serotonergic hallucinogens, making it uniquely advantageous over traditional 5-HT sensor methods and useful for screening additional compounds in vitro for hallucinogenic properties. This also improves through-put screening potential compared to the more time-consuming and costly rodent behavioral assays that assess HTR. In essence, the study demonstrated an effective imaging platform for discriminating hallucinogenic from non-hallucinogenic 5-HT2AR ligands and other psychoactive compounds with low 5-HT2AR affinity. Importantly, the study screened a number of compounds with unknown hallucinogenic potential, and successfully identified a non-hallucinogenic psychedelic analog (‘AAZ-A-154’) which produced neuronal growth and rapid and lasting anti-depressant effects comparable to TBG (Cameron et al. 2021; Dong et al. 2021). Specifically, the compound decreased immobility in the FST and increased sucrose preference in VMAT2-heterozygous mice, resulting in indistinguishable behavior from that of WT controls. This increase in sucrose preference persisted for 12 days.

Ibogaine, itself a non-traditional psychedelic derived from Tabernanthe iboga (an African shrub touted for its therapeutic potential for substance use disorders Noller et al. 2018; Wasko et al. 2018)) has also been explored for developing non-hallucinogenic psychedelics. In contrast to the pharmacology and chemistry of classic psychedelics, ibogaine’s key structural features include an indole ring, a 7-membered tetrahydroazepine, and a bicyclic isoquinuclidine (Iyer et al. 2021; Maciulaitis et al. 2008). Furthermore, ibogaine’s precise mechanism of action is still unclear, with evidence of its action on multiple receptors to mediate its antiaddictive behavioral effects (Wasko et al. 2018). Cameron et al. used principals of function-oriented synthesis to identify the critical motif (indole-fused tetrahydroazepine) responsible for the compound’s psychoplastogenic properties (i.e., the ability to change neurite growth, dendritic spine density, synapse number, and intrinsic excitability, etc.)(Cameron et al. 2021). This paved the development of ‘tabernanthalog’ (TBG), an analogue of ibogaine retaining the psychoplastogenic structural motif and thus the ability to induce neuroplastic changes as demonstrated by increased dendritic growth, dendritic spine density, and spine formation in unstressed rat cortical neurons. Importantly, the development of TBG reduced the number in chemical synthesis to a single step and significantly diminished lipophilicity – the latter a major factor in the cardiotoxic properties of ibogaine. Moreover, through a series of serotonin receptor functional assays, TBG was shown to be relatively selective in 5-HT2AR activation with antagonism displayed at 5-HT2B receptors (chronic stimulation of the latter being implicated in cardiac abnormalities) (Choi & Maroteaux 1996; Choi et al. 1997; Hoelen et al. 2009; Nebigil et al. 2000; Waldum et al. 2022). Having demonstrated potential safety of TBG, the group used the HTR assay to establish non-hallucinogenic potential, with 5-MeO-DMT serving as a positive control. TBG, unlike 5-MeO-DMT, elicited little to no response in this assay. Further support of the non-hallucinogenic potential of TBG stemmed from behavioral response studies in larval zebrafish which, upon treatment with TBG across a range of doses, had behavioral profiles similar to that of the vehicle control. With non-hallucinogenic potential thus established, the critical question of therapeutic efficacy was then addressed. Consistent with previously described observations of ibogaine’s antiaddictive properties (He et al. 2005; Noller et al. 2018), TBG was shown to reduce alcohol- and heroin-seeking behavior through representative murine models. Specifically, an intermittent-access, two-bottle choice experiment, a model for binge-drinking behavior, displayed decreased binge drinking with systemic TBG administration 3 h before a drinking session. Of note, total ethanol intake was lower for at least 48 h after TBG administration, highlighting sustained therapeutic value. Similarly, in a rat model of heroin self-administration, TBG was found to acutely diminish heroin-seeking behavior at three distinct periods: during self-administration, immediately before extinction, or before cued reinstatement. Interestingly, cue-induced relapse was reduced in the TBG groups, an effect observed 12–14 days before cued reinstatement, again highlighting the sustained anti-addictive effects of a single dose of TBG. Although ibogaine is most noted for antiaddictive potential, the investigators also explored its antidepressant potential. For this purpose, the forced swim test (FST) behavior, a measure of behavioral despair in mice and a proxy for activation of medial prefrontal cortical (mPFC) circuits, was assessed. The time spent immobile in this assay after 7 days of unpredictable mild stress was significantly reduced with TBG (50 mg/kg). A direct comparison of TBG with ketamine (known to produce antidepressant-like behavior in the FST in the absence of stress) also revealed reduced immobility at 24 h after treatment with both drugs, but the effects of TBG were not long-lasting (> 24 h). Given the similarity of TBG to ibogaine, this finding is not surprising as the expectation was perhaps greater relief of antiaddictive (versus antidepressive) behavior. Future studies linking TBG with antidepression are warranted. Regardless, the brief spell of antidepressive behavior observed is in line with the psychoplastogenic model whereby increased structural plasticity within the PFC is thought to underlie therapeutic benefits of these compounds (Ly et al. 2018). Furthermore, TBG retained the ability to induce neuroplastic changes as was demonstrated by increased dendritic growth, dendritic spine density, and spine formation in unstressed rat cortical neurons. Lu et al. took this a step further by evaluating these effects of TBG on the stressed brain (Lu et al. 2021). By using a 7-day unpredictable mild stress model on 2-month old mice, they showed that a single TBG dose (10 mg/kg; lower than that used in the previous study thus implying a broader therapeutic window) can rescue deficits at the neuronal (dendritic spine dynamics) as well as behavioral level (anxiety, sensory processing, and cognitive flexibility as assessed by the elevated plus maze, whisker-dependent texture discrimination task, and four-choice odor discrimination and reversal task, respectively).

## Unraveling the evidence

Since the report by Griffiths et al. (Griffiths et al. 2006) of the prospective occasioning of mystical experiences by psilocybin in otherwise healthy volunteers – one of the first methodologically sound human trials of the twenty-first century – a spate of clinical trials investigating psychedelic efficacy across diverse neuropsychiatric conditions have followed. With time, one thing became increasingly clear: classic psychedelics have clinical therapeutic value. Moreover, these compounds have transdiagnostic therapeutic application with lasting impact. Notwithstanding these impressive strides in psychopharmacology – ones that follow a protracted lull in psychedelic research and that arrive against a backdrop of therapeutic stagnation given that current modalities (e.g., SSRIs/SNRIs) fail to adequately treat about 30% of the MDD patient population – the limitations of current clinical trials need emphasis, particularly as they pertain to the question central to this review (Lewis et al. 2019; Trivedi et al. 2006). Perhaps most obvious is that current clinical evidence consists of associations between ASE and enduring therapeutic properties. Although strongly suggestive, these do not equal causation and much remains to be learnt about why such associations exist. Furthermore, the limited sample size and open-label nature of some studies makes drawing inferences difficult. Noteworthy too is that discrepancies in these associations exist between clinical trials, with three aforementioned studies finding no evidence of an association (Carhart-Harris et al. 2021; Sloshower et al. 2023; von Rotz et al. 2023), although the reason for one may be the limited sample size and exploratory nature of the study (Sloshower et al. 2023). Outside of limited recruitment for current psychedelic clinical trials, other challenges include the lack of functional blinding amongst both participants and investigators, strong subject-expectancy effects, the lack of standardized psychotherapeutic interventions and the consequent degree of therapeutic alliance. For extensive reviews on the challenges of psychedelic clinical research, we refer readers to some excellent reviews. (Aday et al. 2022; Muthukumaraswamy et al. 2022, 2021). Muthukumaraswamy has emphasized establishment of causal pathways between treatment and outcome through biomarker-driven causal mediation analyses before ultimate clinical approval of psychedelics (Muthukumaraswamy 2023). We concur with this recommendation and echo a need for capturing the heterogeneity in patient response, whether through pharmaco-imaging approaches, as suggested by some, or with multi-omic tools rooted in quantitative biological measures (Moujaes et al. 2023).

Equally important to acknowledge are the limitations of preclinical studies with psychedelics. For one, the main rodent behavioral surrogate measure of psychedelic ASE (HTR) is an imperfect one. While the HTR displays high predictive validity, it suffers from lack of specificity and has minimal face validity in humans (Canal & Morgan 2012; Carhart-Harris 2023). A similar critique can be made about rodent behavioral despair surrogates (e.g., FST, TST). Separately, significant inter-species differences exist in the 5-HT2AR, such as a single amino acid change (Ser242 and Ala242 in humans and rodents, respectively) that alters the pharmacodynamics of various psychedelic and non-psychedelic compounds and these can limit extrapolation of findings between species (Johnson et al. 1994; Kim et al. 2020). Preclinical studies are also predicated on the assumption that 5-HT2AR activation is the primary means of achieving psychedelic therapeutic benefits but at least one study has cast doubt on that assumption (Hesselgrave et al. 2021). Although the corresponding clinical evidence that ketanserin – a non-selective 5-HT2AR antagonist – blocks most ASE of psilocybin appears suggestive of 5-HT2AR centrality (Vollenweider et al. 1998), ketanserin’s non-selective action on other receptors (5-HT2 family, adrenergic, VMAT, etc.) undermines a causal role of 5-HT2AR in psychedelic action (Gumpper & Roth 2023). Finally, despite the appeal of ‘non-psychedelic psychedelics’ (e.g., TBG, (R)-69/70, Ariadne), until results from human subjects are published, they remain a distant reality, one with tremendous implications but unactualized potential. Additional limitations of psychedelic animal studies are covered elsewhere (Hanks & Gonzalez-Maeso 2013; Pedicini & Cordner 2023).

Notwithstanding these challenges, the psychoplastogenic model that has emerged from animal studies has served the field well (Ly et al. 2018; Vargas et al. 2023). In addition to deepening our understanding of psychedelic mechanism of action, the data have converged on a shared understanding with ketamine’s (another therapeutically effective psychoplastogen) neurobiological effects, particularly with regard to downstream signaling pathways (Aleksandrova & Phillips 2021; Davoudian et al. 2023; Savalia et al. 2021). This raises the question: are the ASE of classic psychedelics uniquely situated to confer persisting benefits when ketamine, with its substantially different ASE, produces durable clinical benefits with ostensibly similar mechanisms of action (i.e., promoting neuroplasticity)? Interestingly, the decision of ketamine dose escalation to achieve increasing dissociative effects – and presumably increased antidepressive effects – is one that currently confronts clinicians. Muddying this decision is conflicting data on the relationship of dissociative effects and therapeutic benefits with clinical trials demonstrating overall superiority of higher doses (0.5–1.0 mg/kg of intravenous (IV) ketamine) but failing to discover significant correlations between the clinician administered dissociative states scale (CADSS) and improvement in depression (Cusin et al. 2017; Fava et al. 2020). Further confounding this relationship are unexpected results from a dose-escalation trial of IV ketamine in treatment-resistant depressed (TRD) patients that found that although improvement during treatment with 0.75 mg/kg was significantly greater than 0.5 mg/kg, depersonalization (a component of CADSS) was experienced in a much higher percentage of patients in the low-dose group (64%) versus the high-dose group (31%) (Cusin et al. 2017). Independently, the psychoplastogenic model has also spurred investigations into molecular adaptations that underlie neuronal changes seen with psychedelic exposure. Interesting observations by Ravenga et al. implicate the role of epigenomic changes in the lasting effects of psychedelics (de la Fuente Revenga et al. 2021). Specifically, they found that certain enhancers regions of the genome remain in an altered state seven days after administration of DOI (2,5-Dimethoxy-4-Iodoamphetamine) (a classic psychedelic) in mice and that these changes outlast the in vivo presence of the compound. Furthermore, these epigenomic alterations were generally of higher magnitude than shorter-term transcriptomic changes.

Additional evidence for the preceding question comes from work by Nardou et al. which offers a novel neurobiological model for the relationship between psychedelic ASE and its sustained therapeutic response (Nardou et al. 2023). Investigating critical periods for social reward learning (CPSRL) – neurologic developmental stages with enhanced sensitivity to environmental stimuli that underlies learning – they first showed that a single dose of MDMA reopens this period in mature adult mice (Nardou et al. 2019). MDMA, an empathogen or non-classical psychedelic, is rapidly approaching FDA clinical approval for PTSD and much of its therapeutic success lies in its prosocial ASE that purportedly aids patients in navigating complex traumatic memories. Given the difference in ASE of MDMA and classic psychedelics and yet similar durable therapeutic responses, the question of whether classic psychedelics may also serve as reopeners of CPSRL was subsequently addressed. Not only were psychedelics found to reopen CPSRL in mice, but the duration of their ASE was also directly proportional to the duration of the open state of CPSRL. Furthermore, this characteristic was generalized across psychoactive agents spanning pharmacologic profiles: LSD and ibogaine with their extended duration of ASE maintained open states longer (3 and 4 weeks, respectively) than psilocybin and MDMA (2 weeks) which, in turn, sustained open states longer than ketamine (2 days). Underlying this ability to reopen CPSRL were metaplasticity mechanisms, forms of higher order synaptic plasticity shown previously to involve oxytocin in the context of social reward learning (Abraham & Bear 1996; Dolen et al. 2013; Hung et al. 2017). Going even further, the group identified transcriptional regulation of the extracellular matrix as an overarching biologic trigger for these metaplastic changes, a property again shared across the tested psychedelic compounds. Rather than undermine the therapeutic value of the ASE – as suggested by the existing preclinical work in this review – findings from this far-reaching study suggest a shared component in the ASE across diverse compounds, one that “embodies the subjective experience of reopening critical periods”. Its clinical relevance cannot be overstated, as it emphasizes the significance of leveraging the post-treatment integration period for clinical benefit.

**Fig. 1:**
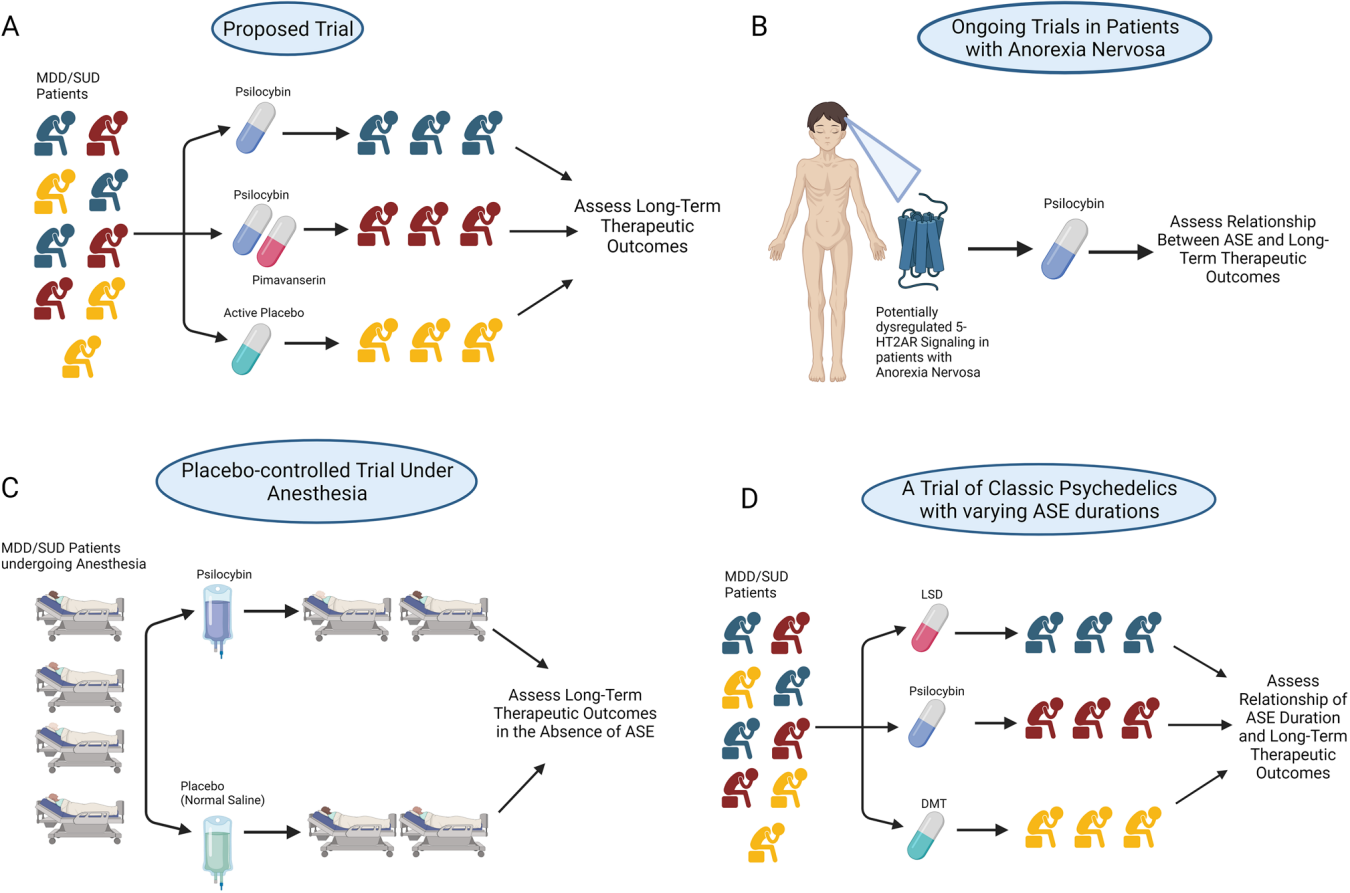
(**A**) A triple-arm placebo-controlled trial in MDD/SUD patients employing psilocybin as well as pimavanserin, a selective 5-HT2A antagonist, in order to eliminate ASE. A robust therapeutic response in the co-administration arm (i.e., psilocybin + pimavanserin) would discount the requirement of ASE for sustained therapeutic benefits of psychedelics. Absence of a response, however, would not necessarily support the necessity of ASE. (**B**) Ongoing trials in patients with anorexia nervosa who have dysregulated serotonergic neurotransmission (particularly at 5-HT2AR) – and presumably blunted ASE – could shed light on the relationship between ASE and long-term therapeutic outcomes (ClinicalTrials.gov, 2023a, 2023b, 2023c) (**C**) A comparison of long-term therapeutic outcomes in MDD/SUD patients who are administered psychedelics with and without general anesthesia. Evidence of lasting therapeutic benefits in anesthetized patients (i.e., lacking ASE or any memory of the acute experience) equaling that of patients in the ‘awake’ arm would undermine the necessity of ASE. (Yaden & Griffiths 2021) (**D**) Classic psychedelics with dramatically reduced durations of ASE (e.g., DMT) could preclude the forms of mystical-type experiences typically associated with longer-acting psychedelics (e.g., psilocybin) and the absence of such experiences could be brought to bear on long-term outcomes

Plausible clinical scenarios exist that could refine the contours of the debate presented in this review and directly address the question (Fig. 1). Microdosing of psychedelics – i.e., the use of sub-threshold doses (typically between a full pharmacological dose and a pharmacological microdose, e.g., 5–10 µg of LSD) to purportedly enhance cognitive and affective measures – could illuminate the dose–response relationship of psychedelic ASE and therapeutic outcomes (Kuypers et al. 2019). Recent clinical trials with repeat low-dose LSD and psilocybin did not find evidence to support therapeutic potential, although a single-low dose of LSD was found to increase reward-related brain activity (Cavanna et al. 2022; de Wit et al. 2022; Glazer et al. 2023; Marschall et al. 2022). For example, the de Wit group randomized healthy young adults to 13 or 26 ug of LSD or placebo across 4 sessions separated by 3–4 days, and did not find evidence of mood improvement or performance enhancement on psychomotor or emotion tasks (de Wit et al. 2022). However, a single low-dose of LSD (13 ug or 26 ug) was enough to increase reward-related brain activity as determined by electrophysiological measures (Glazer et al. 2023). Such dosing regimens are unlike those followed in non-microdosing clinical studies wherein psychedelic therapeutic efficacy is evaluated with a single high-dose (or, at most, two dosing sessions typically separated by weeks) within the context of extensive psychological support. Nonetheless, both regimens share a finite number and a fixed duration of doses and treatment, respectively. In stark contrast are chronic microdosing regimens adhered to and prematurely advocated in non-research settings which involve the daily (or separated by a few days) administration of low-dose psychedelics for an undefined period. Not only may this have attendant risks of cardiac valvulopathy, but there are also other unknowns about such a regimen including the potential for abuse. In general, the evidence from microdosing studies, albeit limited, may suggest the necessity of ASE for sustained therapeutic benefits or, alternatively, could be construed as insufficient pharmacologic activation to confer therapeutic benefits. Other lines of evidence, suggested by Nutt et al. could also help resolve the debate (Nutt et al. 2023). Specifically, if the magnitude or emotional content of the ASE is a mere reflection of drug pharmacodynamic/kinetic parameters (receptor occupation, brain entry, etc.), then clinical trials in patients with anorexia nervosa – a population with potentially dysregulated serotonergic neurotransmission, particularly at 5-HT2AR – could clarify the relationship between ASE and therapeutic outcomes (ClinicalTrials.gov, 2023a, 2023c; Kaye 2008; Spriggs et al. 2021). Indeed, clinical neuroimaging studies have demonstrated reduced binding at 5-HT2AR in distinct brain regions of recovered AN patients compared to healthy control subjects (Audenaert et al. 2003; Bailer et al. 2004; Frank et al. 2002). Although results from the phase 1 clinical trial of psilocybin in AN patients (described earlier) provide little in the way of an ASE correlation with therapeutic outcomes, it is noteworthy that 90% of patients reported that one dosing session was not enough (Peck et al. 2023). Ongoing clinical trials in this patient population will likely provide further answers (ClinicalTrials.gov, 2023a, 2023c). Short-acting psychedelics (e.g., DMT, 5-MeO-DMT) could similarly contribute to this understanding since the temporal abbreviation of their ASE (< 1 h) might preclude the forms of personal insight typically attributed to symptomatic amelioration. Insight on this idea derives from results from the first phase 1 clinical trial of pure DMT (0.1 and 0.3 mg/kg) in MDD patients (n = 7) (D'Souza et al. 2022). D’Souza et al. demonstrated efficacy of 0.3 mg/kg DMT in substantially reducing HAMD-17 scores (4.5 points mean reduction; a medium to large effect size) but no significant correlation between these scores and measures of ASE (with ASC questionnaire and PSI (psychotomimetic states inventory)). Interestingly, participants rated their experience as challenging and intense, yet still meaningful. More clinical work is necessary to map the correlation between the duration of ASE and that of clinical benefits, ideally with long-term studies demonstrating replication. Yaden et al. have suggested the administration of psychedelics to anesthetized individuals so as to determine the necessity of ASE for therapeutic efficacy (Nautiyal & Yaden 2023; Yaden & Griffiths 2021). The problem here, outside of the ethics and enormous costs associated with conducting such a clinical trial, is that anesthetic agents can themselves induce neuroplasticity and have potential antidepressive effects too which could thus confound the effects of the psychedelic. At least one recent clinical trial explored this strategy with ketamine and the results were surprising (Lii et al. 2023). This triple-blinded, randomized, placebo-controlled trial evaluated the effect of ketamine on depression severity in MDD patients undergoing anesthesia for routine surgery and failed to show an effect of group assignment on change in depression severity. Secondary and exploratory outcomes also did not find any benefit of ketamine over placebo. The observed decrease in MADRS scores, although not differing significantly between the two arms, was similar to previous ketamine trials in awake patients. Results of this study highlight the role of subject-expectancy bias in the therapeutic efficacy of drugs with psychoactive effects and the importance of effective blinding. An alternative scenario would be co-administration of psychedelics and benzodiazepines (the latter to suppress subjective awareness) to assess the role of ASE (Nautiyal & Yaden 2023). Comparative pharmacological studies may also be informative. Clinical trials with more selective 5-HT2AR antagonists (e.g., pimavanserin) co-administered with a psychedelic could more conclusively address the ASE requirement for therapeutic benefits (Abbas & Roth 2008). Specifically, if patients in the coadministered arm (i.e., psilocybin + pimavanserin) lack ASE (as expected) and demonstrate long-term symptom improvements, then the requirement for ASE would be called into question. However, a negative therapeutic response in this arm does not necessarily reinforce the ASE requirement given that 5-HT2AR blockade may be preventing the underlying therapeutic mechanism of action. At least one study employed a similar approach where ketanserin was coadministered with psilocybin (Vollenweider et al. 1998). Although ASE was completely abolished by ketanserin, the study was performed in healthy volunteers and thus long-term therapeutic outcomes were not assessed. More recently, a case report of full remission of treatment-resistant depression in a 45-year old man was described in the setting of inadvertent trazodone (a 5-HT2AR antagonist) and psilocybin co-administration (Rosenblat et al. 2023). The patient reportedly lacked all ASE during the dosing session and yet retained improvements in symptoms six months later. A single case report may warrant skepticism but if reproduced in larger trials, the results could impact the question presented herein.

Our review has limitations. For one, it is not a systematic search of all studies conducted with classic psychedelics. Instead, we present the latest advancements of the twenty-first century in the way of clinical and preclinical work to provide readers with a more comprehensive impression on this question. For another, clinical studies center on psilocybin by virtue of the preponderance of studies conducted with the compound and its consequent proximity to clinical approval, although we acknowledge that trials conducted with other classic psychedelics exist and may offer reinforcing or contrasting views on the question.

## Conclusions

The richness of a psychedelic experience, viewed through the lens of countless patient reports, is unlikely, if ever, to be fully captured and reflected by animal behavioral surrogates. Nevertheless, current preclinical work is providing compelling grounds for reevaluating the ASE requirement for neuropsychiatric therapeutic benefits. Not only is this especially relevant to broaden access to and democratize psychedelic-assisted therapy, but also to include patient sub-populations that may otherwise be excluded on medical grounds or other reasons. To be clear, the consideration of ASE necessity for therapeutic benefit discussed here is in relation to neuropsychiatric disorders only and not to other conditions for which psychedelics, irrespective of ASE, may hold potential. But assuming therapeutically effective compounds without these experiences (i.e., ‘non-psychedelic/non-hallucinogenic psychedelics’) do receive eventual clinical approval, their selection as the default therapy remains questionable on ethical grounds. Borrowing a page from the moral ethics of positive morality, Yaden et al. have argued that if traditional psychedelics foster meaning and positive experiences in a patient’s life (often regarded as among the most meaningful experiences of one’s life), then the provision of such experiences may be an obligation for healthcare providers, extending beyond their moral aims of nonmaleficence (“do no harm”) to encompass beneficent duties too (Yaden et al. 2022). In a similar vein, perspectives from hedonistic and consequentialist moral theories have been proposed to inform the justification of pharmacologically-induced meaning in patients (Miceli McMillan 2021). We contend that certain facets of ASE may be instrumental to persisting therapeutic benefits, as already alluded to by some studies (Nikolaidis et al. 2023; Roseman et al. 2017). Perceptual disturbances, for instance, may not be as important as mystical experiences with deep personal meaning for catalyzing sustained behavioral change. With the rapidly advancing front of psychedelic medicine, an answer may not be far.
